# Neuroscience informed risk assessment and management in forensic mental healthcare: a qualitative analysis of ethical and legal challenges and practice-oriented recommendations

**DOI:** 10.3389/fpsyg.2026.1834659

**Published:** 2026-05-28

**Authors:** Yudith Haveman, Vero Jin, Josanne D. M. van Dongen

**Affiliations:** 1 Department of Psychology, Education and Child Studies, Erasmus University Rotterdam, Rotterdam, Netherlands; 2 Amsterdam Public Health Research Institute, Amsterdam UMC, Amsterdam, Netherlands

**Keywords:** ethics, legal issues, neuromodulation, neuroprediction, neurotechnology, risk assessment

## Abstract

Risk assessment for determining the risk for recidivism in offenders, is a core task within forensic mental healthcare. While current tools predict recidivism with 65–75% accuracy, emerging research suggests that neurobiological factors may enhance predictive validity and aid in treatment response and risk management. Yet, translation into forensic mental health practice stays limited. This study explores the ethical and legal considerations of integrating neurotechnology, specifically neuroprediction and neuromodulation, into forensic mental healthcare. A structured conceptual frameworkis provided as input for the qualitative focus group study (*N* = 16) involving professionals and experts by experience. The focus groups revealed a complex interplay between human rights, normative principles, and practical concerns. Key themes include mental integrity, privacy, stigmatization, and informed consent. Participants emphasized the need for contextual interpretation of neural data and transparent communication with clients when neurotechnology, including neural data is used. The findings support cautious optimism for the role of neurotechnology in forensic care, contingent on further research and interdisciplinary dialogue.

## 1 Introduction

Recent studies suggest that neurobiological factors, including dysfunction in brain regions such as the prefrontal cortex, amygdala, anterior cingulate cortex (ACC), and insula, are associated with delinquent behavior (Aharoni et al., 2014; Allen et al., 2022; Boccardi et al., 2011; Darby, 2018; Johanson et al., 2020). Additionally, offenders quite often display brain damage, such as brain injuries or neurological damage, which are partly caused by drug use and alcohol use (Katzin et al., 2020; Kempes et al., 2019) and may explain their violent behavior (Sergiou et al., 2022; Jansen and Franse, 2024; Pickard and Fazel, 2013). Neurotechnology refers to “the field of devices and procedures used to access, monitor, investigate, assess, manipulate, and/or emulate the structure and function of the neural systems of animals or human beings” (International Bioethics Committee, 2022). In criminal justice, neurotechnology is currently mostly used in the form of brain scans to identify neurological disorders (i.e., diagnostic assessments), such as brain damage, neurodegenerative disease, and other neurological conditions. The detected anomalies may be presented in court as evidence and can aid in determining legal insanity and the fitness to stand trial (Del Olmo et al., 2020; Farahany, 2015; Meynen, 2022; Mia, 2025; Morse, 2018).

Besides the use of neurotechnology such as brain scans for clinical or neurological diagnostic purposes, two other applications of neurotechnology are recently discussed, namely the use of neural data in assessing violence risk (i.e., “neuroprediction”), and the use of neurotechnological innovations such as neuromodulation techniques as treatment or rehabilitation intervention (i.e., the management of risk).

In neuroprediction, or neuro-informed risk assessment as we like to call it, the idea is to use neural data obtained from different neuroimaging techniques to enhance existing risk assessment practices by enabling prediction of recidivism risk. For example, studies have shown that lower ACC activity is associated with increased recidivism risk and that adding neurobiological measures, such as heart rate variability, brain activation during inhibition tasks and error-processing measures, to clinical assessments can substantially improve predictive accuracy (Aharoni et al., 2014; Allen et al., 2022; Delfin et al., 2019; Zijlmans et al., 2021; Van Dongen et al., 2025).

Also, neurobiological markers are found to have value in aiding to treatment responsiveness, for instance when managing risk. Recent findings show that alteration of brain activity through neuromodulation can increase neural empathic responding and decrease aggressive behavior and risk taking in offenders (Kuhn et al., 2024; Sergiou et al., 2022; Sergiou et al., 2023). These interventions may result in earlier prison releases and increasing safety and resilience in society once efficacy measures are reliably established (Meynen et al., 2023).

However, the use of neurotechnology in the context of risk assessment and management in forensic practice raises ethical and legal concerns, particularly regarding mental privacy, autonomy, and human dignity (Bijlsma et al., 2022a; Bijlsma et al., 2022b; Ligthart, 2023; Meynen, 2020; Nadelhoffer et al., 2012), and may implicate rights protected under the European Convention on Human Rights. For example, the ability to access or alter thoughts or emotions could undermine individual autonomy, the reliability of testimony, and the integrity of the legal system (Mia, 2025). Addressing these ethical and legal concerns and ensuring that neurotechnologies are efficacious, reliable, and compliant with human rights is essential for their responsible integration into forensic mental healthcare (Dore-Horgan, 2022; Ligthart et al., 2023; Meynen et al., 2023; Geukes et al., 2024).

Therefore, the current study aims to explore ethical and legal challenges of neurotechnology in the defined context of risk assessment and management within forensic mental healthcare. Through a structured theoretical framework and conducting qualitative focus groups with experts, professionals and people with lived-experiences, this study will clarify key challenges and provide guidance for responsible and rights compliant translation of neurotechnology into forensic mental healthcare.

## 2 Theoretical framework

Within this framework, we first discuss neurotechnology, including the techniques and brain structures currently studied, along with ethical and legal considerations such as privacy, autonomy, integrity, and human dignity that apply to any neurotechnology. We then outline neuroprediction and neuromodulation, focusing on their current research, applications, and the ethical and legal challenges specific to their use in forensic mental healthcare.

### 2.1 Neurotechnology

Neurotechnology encompasses any method or electronic device that interfaces with the nervous system to monitor or modulate neural activity. In forensic research, commonly studied techniques include MRI, fMRI, and EEG, which allow visualization of brain structures or record neural activity, captivating anatomy, functional activation and electrical signals, respectively. Although their practical use in criminal law and forensic care remains limited, mostly to assessing neurological conditions, research has identified various subtle brain anomalies linked to antisocial behavior, psychopathy, aggression and delinquent behavior (Cupaioli et al., 2021; Johanson et al., 2020; Marazziti et al., 2013; Saladino et al., 2021; Van Dongen, 2020).

Key brain regions include the amygdala, which responds to threatening and painful stimuli; the ACC, involved in error monitoring and emotional regulation; and the prefrontal cortex, particularly the orbitofrontal cortex, which supports cognitive control. Activity in these areas is typically reduced in individuals with antisocial or psychopathic traits, contributing to aggression and impulsivity (Cupaioli et al., 2021; Johanson et al., 2020; Marazziti et al., 2013; Van Dongen, 2020). Furthermore, psychopathy has also been linked to dysfunction in the default mode network, a system supporting self-referential and moral processing (Johanson et al., 2020; Van Dongen, 2020). Together, these regions play a central role in empathy, honesty, and moral judgment, among other brain areas (Van Dongen, 2020).

While these findings highlight key brain regions underlying behavior relevant to forensic risk assessment and management, their practical application remains limited by ethical and legal considerations, such as privacy, autonomy and mental integrity, and human dignity, which are addressed in the following sections.

#### 2.1.1 Privacy

As neurotechnology develops rapidly, neural data becomes more sensitive due to concerns about a direct link between neural data, cognition and behavior (sometimes referred to as ‘mind reading; Meynen, 2017). The collection of neural data without consent may therefore violate the right to privacy protected under Article 8 of the ECHR, as discussed in recent literature (Bijlsma et al., 2022a; Bijlsma et al., 2022b; Ligthart et al., 2019; Ligthart et al., 2021; Susser and Cabrera, 2023). For that reason, a key distinction must be made between neural data and mental data: neural data primarily reflects brain structure and function, serving as biomarkers for mental states or behavioral tendencies, and should be treated as sensitive personal information, comparable to DNA (Istace, 2022; Ligthart, 2019; Susser and Cabrera, 2023). Conversely, mental data refers to situations in which neural data is interpreted to infer mental processes, such as unconscious decisions, underlying intentions, reactions to visual stimuli, or the withholding of information. The interpretation of neural data in this manner raises concerns regarding freedom of thought and expression under Articles 9 and 10 of the ECHR (Istace, 2022; Trabucco, 2023; Ligthart, 2019; Bijlsma et al., 2022a; Bijlsma et al., 2022b).

These concerns relate not only to access to neural data, but also to the purposes for which it is used and the ways in which it is interpreted (see Ienca and Malgieri, 2022 for a discussion on mental data protection). This is particularly relevant in forensic care, where maintaining confidentiality and trust is essential. However, current forensic research tends to correlate neural data with mental processes in specific contexts. According to the principles of contextual integrity, privacy concerns depend not only on what neural data reveals but also on the context in which it is collected, shared, and interpreted. This highlights the need for clear governance frameworks as neurotechnology and its applications continue to evolve (Susser and Cabrera, 2023; Bijlsma et al., 2022a; Bijlsma et al., 2022b; Ligthart et al., 2019; Ligthart et al., 2021; Trabucco, 2023).

#### 2.1.2 Autonomy and mental integrity

Autonomy and self-determination are core ethical principles, ensuring that individuals can make their own decisions, and is protected under Article 8 ECHR as part of the right to private life. In forensic settings, respect for autonomy implies that a person may refuse the use of neurotechnology for risk assessment, although this could be overridden without infringing Article 8 ECHR as the second paragraph of Article 8 states that autonomy may be restricted when it is necessary for the prevention or detection of crime or for the protection of public safety (Ligthart, 2019). In addition, autonomy is safeguarded in the procedural context by Article 6 ECHR, which protects the right to remain silent and the privilege against self-incrimination, thereby preventing injustice and defending individuals from coercion or oppression by authorities (Ligthart, 2020; Ligthart, 2022). Nevertheless, the voluntariness of consent to neurotechnology remains a major concern, given the inherently coercive nature of criminal law (Pugh, 2018), particularly when its use is incentivized by reduced punishment or other compelling offers (Bijlsma and Ligthart, 2022). Individuals should therefore be adequately informed about its potential consequences (Bijlsma et al., 2022a; Bijlsma et al., 2022b). These issues raise ongoing debate about how legal protections of autonomy should be interpreted and applied in forensic practice (Pugh, 2018; Bijlsma and Ligthart, 2022; Ienca, 2021; Ligthart et al., 2023).

#### 2.1.3 Human dignity

The overarching principle guiding the protection of individuals against the misuse of neurotechnology is human dignity, which requires that persons are treated as ends in themselves rather than as objects (Bijlsma et al., 2022a; Bijlsma et al., 2022b; Bublitz and Merkel, 2014; Moore, 2022). Closely linked to autonomy and mental integrity, human dignity implies that neurotechnology should not undermine individuals’ rational agency or subject them to humiliation or suffering, as prohibited under Article 3 ECHR (Bublitz and Merkel, 2014; Holmen, 2021; Bijlsma et al., 2022a; Bijlsma et al., 2022b). At the same time, it does not necessarily preclude the use of neurotechnology in forensic settings, given that its application may support fair and efficient procedures at some point in time, and may assist the individual to self-development and change (Holmen, 2021; Bijlsma et al., 2022a; Bijlsma et al., 2022b). This requires consistent and transparent implementation, adequate information provision, and safeguards to ensure that individuals’ rights are protected (Bijlsma et al., 2022a; Bijlsma et al., 2022b). These considerations underscore the importance of critically evaluating how neurotechnology is developed and applied in forensic mental healthcare.

### 2.2 Neuroprediction

Current risk assessment tools achieve moderate predictive accuracy (65–75%) and rely primarily on behavioral indicators that indirectly reflect neural functioning (Bogaerts et al., 2018; Fazel et al., 2012; Verweij et al., 2021; Aharoni et al., 2014). Aharoni et al. (2014) were the first to predict recidivism using fMRI, showing that lower ACC activity was associated with higher recidivism risk. Steele et al., (2015) extended this work by adding EEG, achieving 78% accuracy in predicting recidivism. Similarly, Zijlmans et al. (2021) reported accuracy of 68–75%, mainly driven by prior convictions, long-term cannabis use, and reactive aggression. Accuracy increased to 72–80% when biological factors such as processing errors were included. Other studies show that neurobiological measures, such as frontal and amygdala development and regional cerebral blood flow, can improve prediction accuracy up to 82% (Kiehl et al., 2018; Delfin et al., 2019). However, the neurobiology underlying delinquent behavior remains incompletely understood. Nevertheless, these findings suggest that neurobiological data may enhance risk assessment, highlighting the importance of examining the additional ethical and legal concerns associated with its use (van Dongen et al., 2025).

#### 2.2.1 Technical and ethical considerations

Besides its promising contributions, neuroprediction has several limitations. First, neuroimaging is temporally constrained, as it can identify long-term brain abnormalities but not temporary states during an offense, making it difficult to determine onset and causal impact (Bigenwald and Chambon, 2019). This raises questions about responsibility for offenses potentially linked to progressive brain changes and may contribute to reduced responsibility claims, known as the “brain overclaim syndrome” (Gilbert and Focquaert, 2015; Jurjako et al., 2019; Morse, 2005). Moreover, brain abnormalities may create self-fulfilling prophecies and hence can influence behavior, self-perception, and risk assessment as has been seen by disclosing genetic information (Specker et al., 2018; Turnwald et al., 2019). At the same time, brain abnormalities can also help in guiding a treatment target (Tortora et al., 2020).

Second, neuroimaging has interpretive limitations. As most measures are indirect, abnormalities can be associated with behavior but do not establish direct causation, as human agency remains a mediating factor (de Aires Sousa, 2022; Bigenwald and Chambon, 2019; Gkotsi and Gasser, 2016a, 2016b; Morse, 2005). Neuropredictors should therefore be interpreted cautiously and in context, alongside other relevant risk factors (Jurjako et al., 2019; Tortora et al., 2020).

Finally, neuroprediction faces individual application limits. Current accuracy applies primarily to groups rather than individuals, known as the group-to-individual (G2i) inference problem (Aharoni et al., 2022; Nadelhoffer et al., 2012). This may undermine fair trial rights, contribute to discrimination and stigmatization, and conflict with the dignity principle by treating individuals as group members rather than unique persons (Brannan et al., 2019; Jurjako et al., 2019; Tortora et al., 2020; Nadelhoffer et al., 2012). Although this also applies to current risk assessment tools, neuropredictors are only useful if properly translated into practice, taking into account both risk and protective factors, such as treatment possibilities and social support on an individual level (Aharoni et al., 2022; Giordano et al., 2014; Jurjako et al., 2019).

### 2.3 Neuromodulation

Neuromodulation refers to techniques that regulate brain activity of a targeted neuropredictor and consequently may reduce recidivism risk. It can be applied invasively, such as deep brain stimulation (DBS), or non-invasively, such as transcranial direct current stimulation (tDCS) and repetitive transcranial magnetic stimulation (rTMS) (Anselmo et al., 2022). tDCS applies low electrical currents to modulate brain activity and has been associated with reduced aggression in individuals with addiction and borderline personality disorder (Lisoni et al., 2020; Molero-Chamizo et al., 2019; Sergiou et al., 2022; Sergiou et al., 2023; Riva et al., 2015; Riva et al., 2017). Similarly, rTMS uses a magnetic field to alter cortical activity and has been shown to increase empathy and theory of mind in non-forensic populations, although its forensic application remains limited (Anselmo et al., 2022; Balconi and Canavesio, 2013; Schuwerk et al., 2014; Krause et al., 2012). Although neuromodulation shows potential in forensic psychological treatment for reducing aggression and enhancing empathy, further research is needed (Denson et al., 2025). Its use also raises important ethical and legal concerns regarding mental and physical integrity, human dignity, and the right to rehabilitation.

#### 2.3.1 Mental and physical integrity

Neuromodulation can violate autonomy and self-determination if applied without voluntary consent (Douglas, 2014). By altering brain activity via physical intervention, it also implicates bodily integrity, closely linked to the right of privacy (Ligthart, 2023; Tesink et al., 2023). Article 8(2) ECHR allows interference with bodily integrity only if justified for public safety or crime prevention (Ligthart, 2023). Forced neuromodulation must adhere to the principles of subsidiarity, proportionality, and purposefulness (Ligthart, 2023). Non-invasive techniques, such as tDCS and rTMS, cause minimal physical interference and are easier to justify in terms of the principles, but they significantly affect mental processes, empathy, emotions, and subsequent behavior (Anselmo et al., 2022; Sergiou et al., 2022). These interferences can be considered as invasive and therefore be an impeachment on the right of mental integrity according to Article 8 ECHR (Douglas, 2014; Ligthart, 2023). The concept of mental integrity is therefore particularly relevant for neuromodulation in forensic care. However, due to the limited research and case law on neuromodulation in forensic care, the precise meaning, extent, and consequences of this right remain uncertain (Douglas, 2014; Ienca, 2021; Ligthart, 2023).

#### 2.3.2 Right to rehabilitation

Incarcerated individuals may have a right to access neuromodulation for rehabilitation, even given concerns about physical and mental integrity (Bublitz and Merkel, 2014; Dore-Horgan, 2022; Ligthart, 2023). If neuromodulation proves effective for rehabilitation and resocialization, this could be grounded in a moral right to cognitive freedom, or more specifically, neurorehabilitation (Ligthart, 2023). Articles 3 and 5 of the ECHR further support this, emphasizing that offenders should have opportunities for reintegration and personal development while the state is responsible for protecting the vulnerable and minimizing liberty restrictions. Consequently, safe and effective neuromodulation could facilitate rehabilitation while limiting constraints on freedom (Ligthart, 2023).

## 3 Methods

### 3.1 Study design and participants

This study employed a qualitative design, consisting of two focus groups and one individual semi-structured interview to explore ethical and legal issues surrounding neurotechnology, including privacy, autonomy, neuroprediction, and neuromodulation as discussed in the previous chapter. Participants were recruited through the researchers’ professional networks, based on expertise in relevant fields and announcements to sign-up via LinkedIn. To capture diverse perspectives, professionals from various backgrounds were invited, including ethicists (3), those with more legal-ethical background (3), academics (4), clinicians (4), and experts by experience (2). In total, 16 participants took part. Those persons who were part of the research project were excluded from participation in the focus groups. Participants were divided in one focus group of seven, one focus group of eight, and one individual interview. Group composition was determined by professional background and availability. The individual interview was conducted separately at the participant’s request (expert by experience). All participants received written information and provided informed consent.

### 3.2 Data collection

The focus groups and the interview were conducted in Dutch and held in person at Erasmus University, Rotterdam, the Netherlands. The focus groups and interview were conducted by one junior researcher, a master level student and supervised by and associated professor and PI of the study, all with a background in (forensic) psychology and the junior researcher and PI with in addition a background in neuroscience research. At the start of each session, participants were welcomed and provided with an overview of the study’s aims and background. Participants then introduced themselves and shared their initial reflections on the topic. Discussions were moderated to ensure a structured yet open conversation. Each focus group discussed four main topics: neurotechnology in relation to autonomy and privacy, neuroprediction, and neuromodulation. Subtopics were provided under each main topic to structure and guide the discussion. Both focus groups lasted approximately 120 min, and the individual interview lasted 60 min. All sessions were audio-recorded and transcribed verbatim by an external service.

### 3.3 Data analysis

Qualitative content analysis was used to analyze the content of the transcripts (Hsieh and Shannon, 2005; Schreier, 2012) and conducted with use of Atlas.ti (ATLAS.ti Scientific Software Development GmbH, Berlin).

Qualitative content analysis was conducted following the approach of Schreier (2012), combining concept-driven and data-driven strategies in an iterative analytic process. Prior to analysis, a preliminary set of concepts was derived from the exploratory literature framework that informed the topic guide (e.g., privacy as main concept, interpretation and access as second-order concepts; autonomy as main concept, consent and coercion as second-order concepts). These concepts served as an initial orientation rather than a fixed coding template.

After transcription, both researchers independently read all transcripts in full and marked text fragments they considered meaningful in relation to the research question. Differences in the selection and length of marked fragments were discussed until agreement was reached on what constituted a meaningful unit of analysis. Rather than immediately applying predefined codes, both researchers independently sorted these fragments into broad provisional clusters (main concepts and second-order concepts) based on perceived similarities. While these initial clusters partly reflected the concepts from the literature, additional groupings emerged directly from the data, particularly concerning practice-oriented and contextual considerations. Based on these sorted fragments, both researchers independently drafted a preliminary coding scheme, including proposed codes and working definitions. The preliminary codebooks were then compared in detail and merged into a shared coding framework through critical discussion of code scope, wording, and level of abstraction. This process resulted in a codebook specifying main codes, subcodes, and their inclusion criteria.

To explore relationships between codes and to assess whether the coding structure accurately reflected the focus groups, all quotations were arranged visually to examine how they related across clusters. This visual mapping facilitated the identification of patterns, overlaps, and hierarchical relations between codes. As a result, several codes were redefined, merged, or repositioned at different levels of abstraction. For example, concepts initially expected to be central based on the literature (e.g., stigmatization) appeared less prominent in the focus groups, while others (e.g., human dignity and implementation challenges) emerged as more central organizing themes. This cycle of revising the codebook, re-examining the visual overview, and returning to the transcripts was repeated iteratively until the coding structure stabilized and no substantial changes were required. Throughout this process, the researchers continuously checked whether the evolving thematic structure adequately represented the content and emphasis of the focus group discussions.

Finally, all transcripts were re-read in full using the finalized coding scheme to verify contextual accuracy and consistency of theme assignment. Interpretive bias was addressed by independent analytic work, iterative consensus discussions, and by explicitly allowing the data to challenge assumptions derived from the literature framework.

## 4 Results

**Figure 1:**
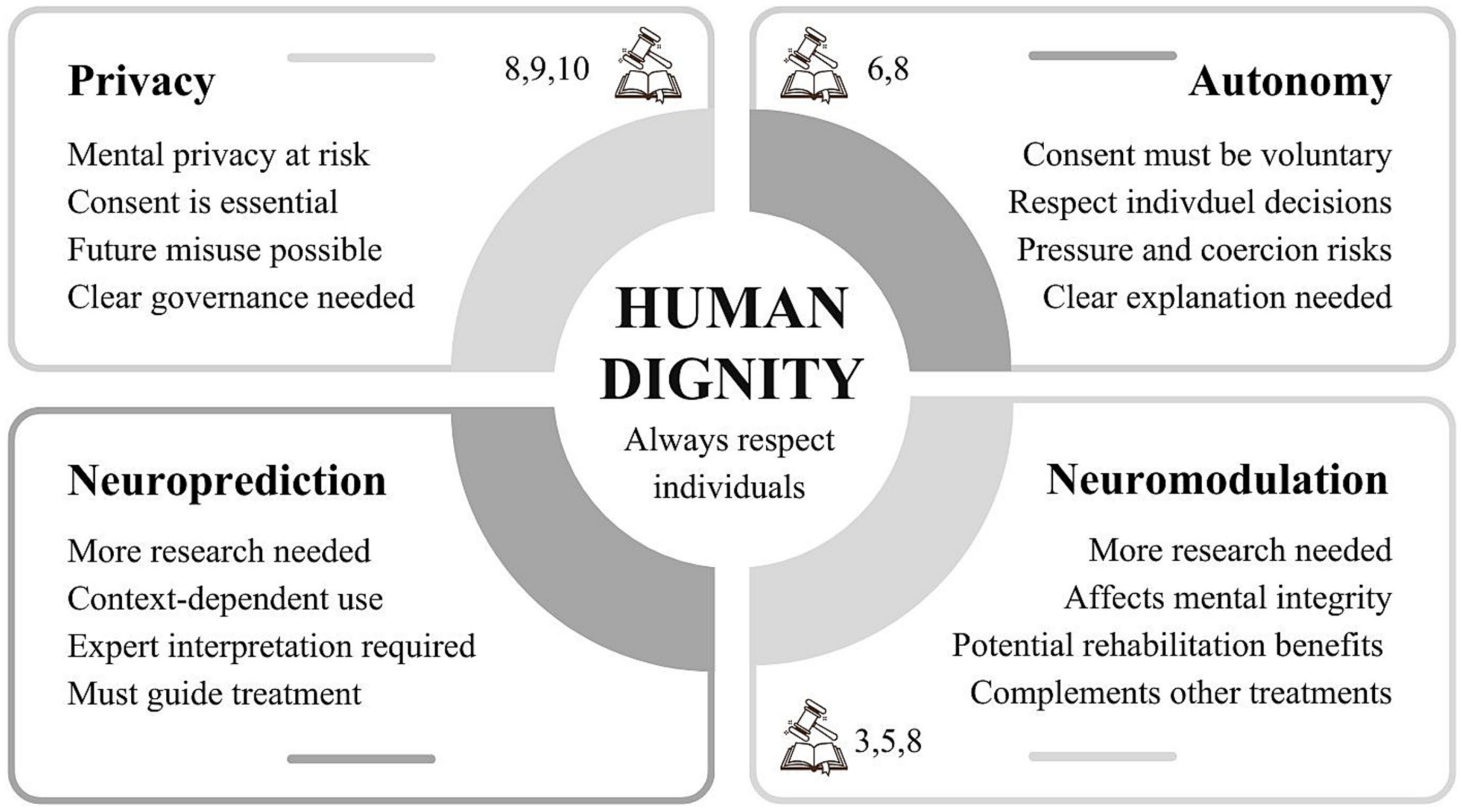
Conceptual overview of key ethical and juridical considerations for using neurotechnology in forensic healthcare, with human dignity as a central guiding principle. Corner icons indicate relevant articles of the European Convention on Human Rights for each theme.

Based on the focus groups, a coding scheme was developed consisting of five main themes (level 1). These were subdivided into 11 subcategories (level 2), which were further specified where necessary (16 categories at level 3 and an additional 9 categories at level 4). It shows that the application of neurotechnology in forensic care is a complex field in which many different concepts are closely interconnected. In the following paragraphs, the categories discussed under each main theme (level 1) are summarized and illustrated using quotes (originally in Dutch but translated into English for publication purposes) from the focus group participants (see also Figure 1).

### 4.1 Neurotechnology

#### 4.1.1 Privacy

“It is always an invasion of privacy, because you, yes, you intrude upon perhaps the most personal thing of someone, namely their own brain, their own thoughts.”

The participants in the focus groups shared the view that certain types of neurotechnology constitute an invasion of privacy if done without valid consent from the person concerned. One academic noted that the acceptability of neurotechnology in relation to similar privacy issues is complex, listing examples: “in a car accident the police may take blood to check whether someone used drugs, and nobody has problems with that; fingerprints may be taken under compulsion; also in the context of pro justitia reports a huge invasion of privacy occurs, your entire life is investigated, and we also consider that acceptable. […] Why do we find one thing completely normal and feel so hesitant about something else, such as neurotechnology?” In short, one stated that people are not yet familiar with neurotechnology, another argued that “it may also be our intuitive idea that neural data contains more of our identity and therefore feels more private.”

Although neuroscientists emphasized that “reading thoughts” is currently not feasible and only possible in very specific experimental contexts, participants expressed concern about future developments. A major shared concern was that “the information you now obtain from a brain scan might initially not be very privacy-sensitive, but if you use the same data 10 years later with the techniques of that time, much more privacy-sensitive information could suddenly be derived from the same data.”

In addition, participants stressed that privacy risks extend beyond data collection to questions of access and data governance. It must be carefully considered who receives such personal information—judges, lawyers, therapists, or others involved in forensic decision-making—as each represents a potential vulnerability in safeguarding confidentiality. Clinicians noted that current infrastructures may not yet be sufficiently robust to guarantee adequate protection of someone’ privacy.

#### 4.1.2 Autonomy

It is always about checks and balances. If someone does not want to participate in research, you must respect that. You could then say to that person: “Listen, based on what you have done and what we see, we believe you cannot be released until we have obtained an expert assessment of your risk of danger. So, I think you must guarantee the autonomy and integrity of the patient in all cases. That relates to human dignity, but it may have consequences.”

This quote from a practitioner reflects a nuanced view of autonomy and human dignity. Academics, practitioners, and experiential experts unanimously agreed that at minimum consent must be granted before a brain scan is taken for risk assessment. However, an academic argued that “the entire risk assessment is a cost–benefit analysis […] and at some point you must make a legal judgment: is this important enough to justify the intrusion [on privacy]?”

“You can of course also see the positive side. If it can really help and lead to faster release or prevent recidivism… but I do think someone must be able to make that decision in a free context and fully understand what they are choosing.” At the same time, concerns were expressed that individuals may feel pressured to consent. An experiential expert estimated that “the fear that it could be used against someone is greater than the hope [that neurotechnology brings].” It is therefore crucial that the limitations and consequences of neurotechnology are clearly explained and under what conditions they apply. For individuals who cannot fully understand this—such as those with intellectual disabilities or certain psychological conditions—the legal system might need a structure, “perhaps in the form of a third committee that reviews it, to ensure a proper assessment and free choice.”

#### 4.1.3 Human dignity

“I understand that we must protect people, but I struggle greatly with the idea that once someone has done something wrong, they are placed under a magnifying glass of a risk assessment instrument, and it feels to me that with this step we increasingly stop seeing the person behind the offense.”

This concern, voiced particularly by experiential experts, reflects the fear that neuroprediction may reduce individuals to risk profiles rather than recognizing them as persons with inherent worth. Questions of equality were closely linked to this concern. As one participant noted: “if you want to do it fairly, you should actually scan everyone [e.g. for pedophilia],” and an experiential expert added: “… and then you must also apply the consequences of that to everyone.” Such remarks illustrate the view that selectively subjecting offenders to predictive neuro-assessments risks undermining the principle of equal moral status.

Participants further warned that neurotechnology may remove behavior from its broader personal and social context, thereby labeling individuals in ways that foster stigmatization and discrimination. Such a label can contribute to a self-fulfilling prophecy: “Yes, I cannot help it. I am my brain. My brain does that,” as one academic phrased it. Whether this differs from general pro justitia reports is debated by some academics, but there is concern that an opaque prediction model increases the risk of stigmatization.

Finally, an academic emphasized that “the outcomes of neuroscans must be linked to some notion of changeability, perspective, or intervention, otherwise you are on a hopeless path that results in stigmatization that helps no one.” Across discussions, safeguarding human dignity was associated with preserving recognition of the individual as more than their risk profile and as capable of change.

### 4.2 Overall results on neuroprediction

The central question across focus groups was the added predictive value of neuroprediction compared with existing risk assessment instruments. Estimates ranged from marginal gains to 10–15%. The clinician who mentioned the 10–15% added, “we are not our brain, but it does produce,…” However, participants emphasized that current scientific evidence remains limited. As one academic noted regarding a specific brain region identified in research: “using something like that as a predictor is a second step. And I do not think we currently know enough to do that in a proper way.”

A recurring concern was what exactly a neuropredictor measures. Does a neuro scan, for example, predict whether someone will actually translate thoughts into actions and thereby predict risk of recidivism? Or is the neuropredictor more a general measure of impulsivity or stress sensitivity? As one academic remarked, “impulsivity, stress tolerance, and other general factors can also be assessed through behavior; such a sophisticated measurement is not necessary.” Participants stressed that any neuropredictor must relate clearly to a specific behavioral outcome, since “a potential neuropredictor must in all cases relate to a certain behavior [i.e., recidivism], but even that may not be sufficient.” because of other individual factors (including protective factors).

Once again, the context in which a neuropredictor is used proved crucial in both focus groups. Neuroprediction was therefore widely regarded as unsuitable as a standalone indicator, and experts who can clearly explain how neuropredictors should be interpreted, are necessary. One participant summarized it follows: “Do not make it a decisive factor, but make it part of the overall profile of a person. And a great deal of professionalism and a strong ethical compass are required to combine all these elements and truly decide: ‘Well, this patient should probably not be released for the time being.”

Besides these concerns, an important positive aspect of neuroprediction was highlighted: “neuroprediction can provide more insight into the underlying mechanisms of behavior. The same behavior can arise in very different ways.” It was further noted that “if you want to address something and you approach it all in the same way while the origin differs, treatment will not be very effective.” The idea of linking neuroprediction to changeability in treatment received strong support among academics, practitioners, and experiential experts. A practitioner responded enthusiastically: “I really see this as the promising aspect of such research; it offers possibilities to better explain and therefore also treat things.” Thus, the role neurotechnology can play in explaining behavior and tailoring treatment may serve as a starting point for its application.

### 4.3 Overall results on neuromodulation

As discussed in the theoretical framework, there is potential in using neuroprediction, provided that the predictor is also linked to changeability and treatment (neuromodulation). Participants of the focus groups viewed this with curiosity; one academic stated that “with proper guidance alongside such treatment, it might reduce recidivism […] and benefit both society and the individual.” Experiential experts emphasized that individual benefit is a critical consideration in light of human dignity.

Debates in the focus groups centered on whether neuromodulation should be voluntary or could be imposed like compulsory medication. Opinions differed: an academic argued that if someone chooses it and believes it could help, it should be allowed. However, the question remained: “can they make a well-informed decision, […] or are they being steered toward the perceived benefit?” In forensic psychiatric settings, where treatment compliance is often required, participants questioned whether true free choice is feasible. Comparisons were drawn to medication: “if compulsory medication is permitted [(i.e., under certain conditions)], similar rules should apply—assuming it produces comparable effects. Why would one be allowed and the other not?”

Participants highlighted multiple uncertainties regarding neuromodulation, including potential permanent changes to character, unintended personality effects, and responses among individuals unfamiliar with neuroscience. As noted, “you cannot predict in advance how someone will respond,” and “psychotherapy and medication also lead to lasting changes.” Benefits were recognized, particularly its potential as a stabilizing intervention: “If someone is swinging from one extreme to another, cognitive behavioral therapy does not work well, so medication is used to make the person more receptive… neuromodulation could have great potential in that respect, perhaps instead of medication, which can be like using a cannon to hit a fly.”

Given limited evidence, participants agreed that neuromodulation should be offered only on a voluntary basis for now. One practitioner summarized: “The first thing it makes me think is: yes, provided the patient gives consent. […] Someone mentioned a moral compass earlier. Like all the treatments we offer that can be intrusive, I see no reason not to offer it. […] I think it is always better to have a package of interventions so you can see what fits best, and the individual decides whether they want to receive it or not.”

### 4.4 Changes in participants’ positions after the focus group discussion

Before the discussion, participants were cautious about neuroprediction in forensic care, questioning its accuracy, risks of reductive or deterministic interpretations and many considerations for implementation were meant. After the focus groups, participants recognized both challenges—that somewhat differed from before the discussion started—and potential benefits. They highlighted the need for research, clear communication, integration with other factors and regulatory guidance in translating neuropredictors accurately into practice. As one participant summarized: “legally there may be fewer objections [to applying neurotechnology] than practical problems regarding ethical principles.”

Participants also highlighted the potential of neurotechnology significantly, particularly the use of neuroprediction to guide neuromodulation. As one experiential expert reflected: “I had resistance. I have somewhat less resistance. There are still many challenges [.] but it could potentially have positive effects on individuals. However, much work needs to be done before it becomes truly applicable.” Overall, the multidisciplinary exchange of knowledge during the focus groups was considered valuable and essential for shaping the future application of neurotechnology in forensic settings.

## 5 Discussion

The aim of this report is to gain insight into the ethical and legal dilemmas associated with the use of neurotechnology in forensic care, and how professionals from different disciplines approach these dilemmas. The literature review showed that the ethical and legal dilemmas surrounding neurotechnology are highly complex. On the one hand, there are rights that favor restraint and protection of the suspect, such as Article 8 of the ECHR (right to privacy). On the other hand, there are normative principles that support a more positive position toward neurotechnology, such as the principle of resocialization. Both the literature review and the qualitative research indicate that the “neural dimension” adds a new layer to existing risk factors and treatments, which also raises concerns. According to focus group participants, this is linked to uncertainty about what information can be revealed through imaging techniques, what information is actually stored in the brain, and how a brain scan should be interpreted and translated into risk assessment in forensic practice, as well as how such information should be handled.

Ethical considerations primarily concern the risk of stigmatization arising from predictive models operating at group level. Participants acknowledged, however, that the same issue applies to current risk assessment practices. The focus groups emphasized that neuroprediction should always be weighed in the context of other variables (such as psychological and social factors), not only to prevent stigmatization but also to safeguard human dignity. Furthermore, obtaining consent for brain scanning—whether for neuroprediction or neuromodulation—was identified as a crucial issue for the application of neurotechnology in criminal justice. The advantages, disadvantages, and consequences of neurotechnology in risk assessment should be communicated transparently to the patient so that an informed and voluntary choice can be made. Finally, linking neuroprediction to neuromodulation as a supportive or stabilizing intervention generated cautious optimism. Further research into both neuroprediction and neuromodulation is required to gain a more nuanced understanding of how neurotechnology relates to legal and ethical principles. For example, it is important to examine whether the benefits of incorporating neurobiological measures into risk assessment (such as brain scans) outweigh the associated costs. In addition, particularly in the Netherlands, it is essential to study how judges handle neurobiological information presented in court.

A frequently mentioned challenge regarding the application of neurotechnology in risk assessment was the potential for stigmatization. Reducing individuals to certain data points and using that information to estimate recidivism risk carries a risk of stigmatization. However, the same problem exists in current risk assessment practices. For example, the leading risk assessment tool currently used in forensics mental health practice is the third version of the Historical, Clinical Risk (HCR-20^v3^) (Douglas et al., 2013) assigns scores to different factors across domains, and the total score—together with clinical judgment—guides the risk assessment. Thus, data reduction and the associated risk of stigmatization are also present in traditional risk assessment. Generally, the risk of stigmatization decreases when risk assessments become more accurate, which can be achieved by incorporating more variables into the assessment. From this perspective, there is merit in adding neurobiological measures, provided they are contextualized alongside existing factors. However, it should be noted that more data may also reduce transparency. Advanced analytical techniques such as machine learning rely on algorithms that produce outcomes, but the underlying factors and interactions often remain unclear. Therefore, algorithms must be rigorously tested before implementation. Beyond incorporating neurotechnology into actuarial risk assessment, it may also be useful for explaining underlying behavioral mechanisms by constructing a biopsychosocial profile (see De Ruigh et al., 2021; Jansen, 2022). The same behavior can arise through different etiological pathways, which cannot always be determined using current risk assessment and clinical judgment. Neurotechnology may therefore contribute to understanding etiology and identifying treatment targets.

Focus group discussions highlighted the importance of precisely understanding what a brain scan does and does not reveal, and which interpretations are justified. This remains challenging. Based on recidivism prediction studies by Aharoni et al. (2014), Allen et al. (2022), Steele et al., (2015), and Zijlmans et al. (2021), the ACC appears to be a promising brain region. However, changes in the ACC are also observed in various psychological disorders such as depression, anxiety and addiction (Akdoğdu and Erbaş, 2021). Thus, the meaning of increased or decreased ACC activity remains unclear, particularly in terms of how such findings should be communicated to judges without implying determinism. This concern also applies to current risk assessment practices, but neurotechnology—being more distant from observable behavior and perceived as a “hard science”—requires especially careful communication.

Another critical issue concerns the predictive value of neuroprediction compared with traditional risk assessment. Participants repeatedly questioned what neuroprediction actually adds, and the answer influences the ethical and legal considerations surrounding its use. Neurotechnology is often scrutinized more strictly than other risk factors, despite the absence of detailed knowledge about the contribution of individual factors within instruments such as the HKT-R or HCR-20^v3^ (Douglas et al., 2013; Spreen et al., 2014). Nevertheless, empirical studies suggest that neurobiological measures can improve recidivism prediction by 5–20% compared to traditional risk factors (e.g., Delfin et al., 2019; Zijlmans et al., 2021). The literature also reflects uncertainty regarding neurotechnology’s role in risk assessment. Some studies emphasize that neural data reflect electrical or magnetic brain activity and cannot currently establish causal links to behavior or cognition (Hallinan et al., 2014; Ryberg, 2017; Susser and Cabrera, 2023; Trabucco, 2023). Other studies warn that future technological developments might eventually enable such causal interpretations (Istace, 2022; Trabucco, 2023). Thus, the central issue may not be the magnitude of neuroprediction’s contribution, but rather the need for careful handling and interpretation of neurological data.

Regarding the autonomy of suspects and offenders, focus group participants stressed that it should be respected as far as possible. The literature review confirmed that individuals have a legal right to privacy and that autonomy and integrity are important ethical values. Although this right can be limited by law, consent currently appears to be the norm in neurotechnology research. However, it remains important to consider the implications for autonomy if neurobiological measures make risk assessments, for example, 10% more accurate. In principle, suspects may refuse participation in research or treatment. Yet if risk assessment becomes increasingly precise and an individual refuses cooperation, what consequences should follow? Guidelines for obtaining informed consent in a free and transparent context are needed. Professionals emphasized that the advantages and disadvantages of neurotechnology must be clearly explained to the client, and that interpretations and consequences should be documented for both the individual and the court.

Furthermore, if neuroprediction can be linked to treatment—an idea that received broad support and may represent the preferred application—the question arises whether true voluntariness is possible. Both literature and participants expressed concern about a “coercive offer,” since a treatable neuropredictor could influence forensic outcomes. However, this issue is not unique to neuromodulation; current treatment options may also feel coercive. The question also arose whether neuromodulation could be imposed like medication. Legally, such compulsion would interfere with physical and mental integrity, protected under Article 8 of the ECHR (Ligthart, 2019). While interference with physical integrity may be justified under Article 8(2), justification for interference with mental integrity is less clear (Ligthart, 2019). Research has not yet established long-term effects of neuromodulation, though direct side effects appear limited to temporary sensations such as burning, tingling and itching (Sergiou et al., 2022). Medication likewise affects the brain and may have long-term effects, yet compulsory treatment is considered permissible. Professionals unfamiliar with neuroscience often perceived this discrepancy as problematic, and practical challenges—such as movement during scanning—further complicate implementation in a coercive setting. Nevertheless, there was support for using neuromodulation to enhance treatment responsiveness. Determining how such applications fit within existing legal frameworks and ethical principles of autonomy and dignity remains a task for multidisciplinary investigation, should research confirm its benefits.

### 5.1 Strengths and limitations

The strength of the focus groups lies in the dialogue between professionals from different disciplines. This approach not only provides an integrated perspective on the issue at hand (as also noted by Bijlsma et al., 2022a; Bijlsma et al., 2022b), but may also contribute to strengthening connections between the various stakeholders involved. Participants indeed indicated that they experienced the focus groups as valuable. Moreover, the thematic structure of the discussions enabled us to obtain broad insights into how different professionals perceive neurotechnology within forensic mental healthcare.

At the same time, participants were recruited through the authors’ professional networks, which makes the sample inherently selective. Additionally, the contribution of “experts by experience” was limited. This is particularly important given that autonomy and coercion are central themes of the paper and are likely most relevant to that subgroup. This selection of participants is particular relevant to mention, as it substantially affects the interpretability and generalizability of our findings. Individuals with particularly strong opinions (and a willingness to express them), or those with specific expertise or curiosity regarding the topic, may have been more inclined to respond to the invitation. Consequently, the sample may not be representative of all stakeholders involved in the forensic field. Alternative perspectives or unexpressed views—potentially constrained by the non-anonymous setting of a focus group—may therefore be underrepresented Also, because we adopted a thematic strategy, the classification process may have been subject to bias. Prior knowledge that the we as researchers may had could have influence coding decisions and may have led to interpretative choices that other researchers might not have made. Nevertheless, we remained open to alternative labels beyond the predefined thematic framework. Although the data could be re-coded by an independent third reviewer, we do not expect that this would substantially alter the results as presented.

With regard to the organization of the focus groups, it would have been beneficial to conduct a pilot session beforehand. This would have allowed us to become more familiar with the methodology and to evaluate the impact of a structured versus a more open discussion format. An evaluation was conducted between the two focus groups to assess possible improvements, both in terms of content and execution. Based on this reflection, we decided to adopt a more open format for the second focus group. However, methodological consistency between sessions is generally preferable, and a pilot group could have facilitated this.

### 5.2 Implications and recommendations

It is evident that the application of neurotechnological innovations in forensic mental healthcare involves a complex interplay of possible advancements and ethical and legal considerations that need to be taken into account.

**Figure 2:**
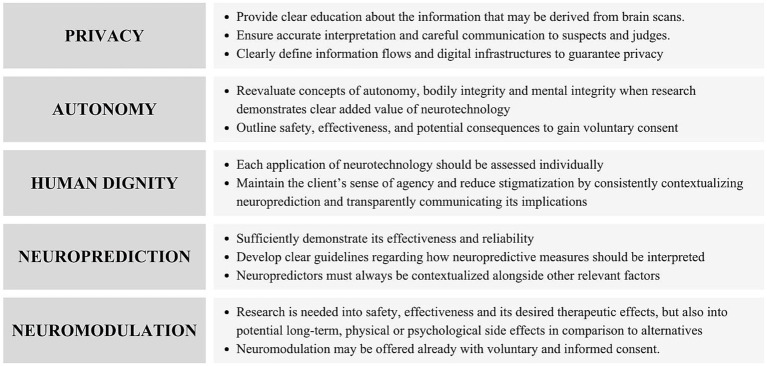
Implications and recommendations for the use of neurotechnology within forensic mental healthcare.

However, even within the constraints of legal and ethical boundaries, it is possible to identify key factors that are essential for granting neurotechnology a more nuanced and carefully considered role within criminal law and forensic mental health practice. It is recommended to carefully document the support for neurotechnological applications before implementing particular technologies into practice. When implementing, the implementation process needs to be carefully planned, organized and monitored to assure legal and ethical principles are taken into account. To accomplish this, we recommend to develop guidelines for the implementation and use of neurotechnology in forensic mental health settings. Recently, an international consortium and Special Interest Group of the International Association of Forensic Mental Health Services was established named FORNEUROTECH. One of its aims is to develop ethical-legal guidelines for (neuro) technological advancement in forensic mental health. See Figure 2 for implications and recommendations regarding the use of neurotechnology.

## 6 Conclusion

This study reviewed the current state of the art regarding the use of neurotechnological innovations in forensic mental health and thematically analyzed current challenges with respect to its responsible use in terms of judicial and ethical considerations. Outcomes of this study showed that neurotechnological innovations may be promising in advancing forensic mental health practice, but that significant steps remain before neurotechnology can be implemented and used in a responsible way. Progressing implementation requires both neuroscientific advancement and a careful expansion and refinement of legal and forensic frameworks. Future research should continue on the use of neural data and neurotechnology in the context of neuro-informed risk assessment and risk management including treatment strategies to support its usefulness and added benefit for forensic metal healthcare. Crucially, this process should occur in a transdisciplinary dialogue with forensic clients, legal professionals, ethicists, academics, and clinicians to ensure responsible interpretation, integration, implementation, and communication of this inherently multidisciplinary field.

## Data Availability

The raw data supporting the conclusions of this article will be made available by the authors, without undue reservation.

## References

[ref1] Aharoni E. Abdulla S. Allen C. H. Nadelhoffer T. (2022). Ethical Implications of Neurobiologically Informed Risk Assessment for Criminal Justice Decisions. Camebridge, Massachusetts: MIT Press. 36095110

[ref2] Aharoni E. Mallett J. Vincent G. M. Harenski C. L. Calhoun V. D. Sinnott-Armstrong W. . (2014). Predictive accuracy in the neuroprediction of rearrest. Soc. Neurosci. 9, 332–336. doi: 10.1080/17470919.2014.907201, 24720689 PMC4059067

[ref3] Akdoğdu B. S. Erbaş O. (2021). Subgenual anterior cingulate cortex and psychiatric disorders. Demiroglu Sci. Univ. Florence Nightingale J. Transplant. 6, 45–51. doi: 10.5606/dsufnjt.2021.025

[ref4] Allen C. H. Aharoni E. Gullapalli A. R. Edwards B. G. Harenski C. L. Harenski K. A. . (2022). Hemodynamic activity in the limbic system predicts reoffending in women. NeuroImage. Clin. 36:103238. doi: 10.1016/j.nicl.2022.103238, 36451349 PMC9668656

[ref5] Anselmo A. Lucifora C. Rusconi P. Martino G. Craparo G. Salehinejad M. A. . (2022). Can we rewire criminal mind via non-invasive brain stimulation of prefrontal cortex? Insights from clinical, forensic and social cognition studies. Curr. Psychol. 42, 20765–20775. doi: 10.1007/s12144-022-03210-y, 35600259 PMC9107958

[ref6] Balconi M. Canavesio Y. (2013). High-frequency rTMS improves facial mimicry and detection responses in an empathic emotional task. Neuroscience 236, 12–20. doi: 10.1016/j.neuroscience.2012.12.059, 23357112

[ref7] Bigenwald A. Chambon V. (2019). Criminal responsibility and neuroscience: no revolution yet. Front. Psychol. 10:1406. doi: 10.3389/fpsyg.2019.01406, 31316418 PMC6610327

[ref8] Bijlsma J. Franken S. van Kampen P. (2022a). “Altijd een komma, nooit een punt?” in Reclassering, Toezicht en Resocialisatie, (Abingdon, Oxon: Wolters Kluwer), 191–274.

[ref9] Bijlsma J. Geukes S. H. Meynen G. Raemaekers M. A. H. Ramsey N. F. Simon Thomas M. A. . (2022b). “Kansen en risico’s van de toepassing van neurotechnologie in het strafrecht,” in Wetenschappelijk Onderzoek- en Documentatiecentrum, (Utrecht: WODC).

[ref10] Bijlsma J. Ligthart S. L. (2022). Medische interventies ter preventie van recidive: Over vrijwilligheid en de houdbaarheid van het onderscheid tussen dwang en drang. Rechtsgeleerd Mag. Themis 2, 41–52.

[ref11] Boccardi M. Frisoni G. B. Hare R. D. Cavedo E. Najt P. Pievani M. . (2011). Cortex and amygdala morphology in psychopathy. Psychiatry Res. Neuroimaging 193, 85–92. doi: 10.1016/j.pscychresns.2010.12.013, 21676597

[ref12] Bogaerts S. Spreen M. Ter Horst P. Gerlsma C. (2018). Predictive validity of the HKT-R risk assessment tool: two and 5-year violent recidivism in a Nationwide sample of Dutch forensic psychiatric patients. Int. J. Offender Ther. Comp. Criminol. 62, 2259–2270. doi: 10.1177/0306624X17717128, 28658999 PMC5960839

[ref13] Brannan C. Foulkes A. L. Lázaro-Muñoz G. (2019). Preventing discrimination based on psychiatric risk biomarkers. Am. J. Med. Genet. B Neuropsychiatr. Genet. 180, 159–171. doi: 10.1002/ajmg.b.32629, 29633550 PMC6173986

[ref14] Bublitz J. C. Merkel R. (2014). Crimes against minds: on mental manipulations, harms and a human right to mental self-determination. Crim. Law Philos. 8, 51–77. doi: 10.1007/s11572-012-9172-y

[ref15] Cupaioli F. A. Zucca F. A. Caporale C. Lesch K. P. Passamonti L. Zecca L. (2021). The neurobiology of human aggressive behavior: neuroimaging, genetic, and neurochemical aspects. Prog. Neuro-Psychopharmacol. Biol. Psychiatry 106:110059. doi: 10.1016/j.pnpbp.2020.110059, 32822763

[ref16] Darby R. R. (2018). Neuroimaging abnormalities in neurological patients with criminal behavior. Curr. Neurol. Neurosci. Rep. 18:47. doi: 10.1007/s11910-018-0853-3, 29904892

[ref17] de Aires Sousa S. (2022). Connections (and limits) between law and natural sciences: the concepts of causality and culpability from the perspective of criminal law. Int. J. Semiot. Law 35, 287–296. doi: 10.1007/s11196-020-09788-5

[ref18] De Ruigh E. L. Bouwmeester S. Popma A. Vermeiren R. R. J. M. van Domburgh L. Jansen L. M. (2021). Using the biopsychosocial model for identifying subgroups of detained juveniles at different risk of re-offending in practice: a latent class regression analysis approach. Child Adolesc. Psychiatry Ment. Health 15:33. doi: 10.1186/s13034-021-00379-1, 34158097 PMC8218478

[ref19] Del Olmo B. R. Murtra P. V. Roig M. À. P. Pozón S. R. (2020). Uses and abuses of neuroscience technology in the courtroom. “I did not do it. It was my brain…”. Ramon Llull J. Appl. Ethics 11, 33–57.

[ref20] Delfin C. Krona H. Andiné P. Ryding E. Wallinius M. Hofvander B. (2019). Prediction of recidivism in a long-term follow-up of forensic psychiatric patients: incremental effects of neuroimaging data. PLoS One 14:e0217127. doi: 10.1371/journal.pone.0217127, 31095633 PMC6522126

[ref21] Denson T. F. Choy O. Summerell E. Wong I. (2025). A meta-analysis of the effects of transcranial direct current stimulation on anger and aggression. Aggress. Behav. 51:e70036. doi: 10.1002/ab.70036 40474608 PMC12141985

[ref22] Dore-Horgan E. (2022). Do criminal offenders have a right to neurorehabilitation? Crim. Law Philos. 17, 429–451. doi: 10.1007/s11572-022-09630-y 37266329 PMC10229454

[ref23] Douglas T. (2014). Criminal rehabilitation through medical intervention: moral liability and the right to bodily integrity. J. Ethics 18, 101–122. doi: 10.1007/s10892-014-9161-6, 25009441 PMC4083266

[ref24] Douglas K. S. Hart S. D. Webster C. D. Belfrage H. (2013). HCR-20V3: Assessing risk of Violence – User guide. Burnaby, Canada: Mental Health, Law, and Policy Institute, Simon Fraser University.

[ref25] Farahany N. A. (2015). Neuroscience and behavioral genetics in US criminal law: an empirical analysis. J. Law Biosci. 2, 485–509. doi: 10.1093/jlb/lsv059, 27774210 PMC5034387

[ref26] Fazel S. Singh J. P. Doll H. Grann M. (2012). Use of risk assessment instruments to predict violence and antisocial behaviour in 73 samples involving 24 827 people: systematic review and meta-analysis. BMJ 345, 1–12. doi: 10.1136/bmj.e4692, 22833604 PMC3404183

[ref27] Geukes S. H. Bijlsma J. Meynen G. Raemaekers M. A. H. Ramsey N. F. Thomas S. . (2024). Neurotechnology in criminal justice: key points for neuroscientists and engineers. J. Neural Eng. 21:013001. doi: 10.1088/1741-2552/ad1785 38193322

[ref28] Gilbert F. Focquaert F. (2015). Rethinking responsibility in offenders with acquired paedophilia: punishment or treatment? Int. J. Law Psychiatry 38, 51–60. doi: 10.1016/j.ijlp.2015.01.007, 25725545

[ref29] Giordano J. Kulkarni A. Farwell J. (2014). Deliver us from evil? The temptation, realities, and neuroethico-legal issues of employing assessment neurotechnologies in public safety initiatives. Theor. Med. Bioeth. 35, 73–89. doi: 10.1007/s11017-014-9278-4, 24442931

[ref30] Gkotsi G. M. Gasser J. (2016a). Critique of the use of neuroscience in forensic psychiatric assessments: the issue of criminal responsibility. Évol. Psychiatr. 81, e25–e36. doi: 10.1016/j.evopsy.2015.10.006

[ref31] Gkotsi G. M. Gasser J. (2016b). Neuroscience in forensic psychiatry: from responsibility to dangerousness. Ethical and legal implications of using neuroscience for dangerousness assessments. Int. J. Law Psychiatry 46, 58–67. doi: 10.1016/j.ijlp.2016.02.030, 27209602

[ref32] Hallinan D. Schütz P. Friedewald M. De Hert P. (2014). Neurodata and neuroprivacy: data protection outdated? Surveil. Soc. 12, 55–72. doi: 10.24908/ss.v12i1.4500

[ref34] Holmen S. J. (2021). Respect, punishment and mandatory neurointerventions. Neuroethics 14, 167–176. doi: 10.1007/s12152-020-09434-8

[ref35] Hsieh H. F. Shannon S. E. (2005). Three approaches to qualitative content analysis. Qual. Health Res. 15, 1277–1288. doi: 10.1177/1049732305276687, 16204405

[ref36] Ienca M. (2021). On neurorights. Front. Hum. Neurosci. 15:701258. doi: 10.3389/fnhum.2021.701258, 34630057 PMC8498568

[ref37] Ienca M. Malgieri G. (2022). Mental data protection and the GDPR. J. Law Biosci. 9:lsac006. doi: 10.1093/jlb/lsac006 35496983 PMC9044203

[ref38] International Bioethics Committee (2022). Ethical Issues of Neurotechnology. Paris, France: UNESCO Publishing.

[ref39] Istace T. (2022). Neurorights: the debate about new legal safeguards to protect the mind. Issues Law Med. 37:95. 36629792

[ref40] Jansen L. M. C. (2022). The neurobiology of antisocial behavior in adolescence; current knowledge and relevance for youth forensic clinical practice. Curr. Opin. Psychol. 47:101356. doi: 10.1016/j.copsyc.2022.101356, 35687917

[ref41] Jansen J. M. Franse M. E. (2024). Executive functioning in antisocial behavior: a multi-level systematic meta-analysis. Clin. Psychol. Rev. 109:102408. doi: 10.1016/j.cpr.2024.102408, 38430781

[ref42] Johanson M. Vaurio O. Tiihonen J. Lähteenvuo M. (2020). A systematic literature review of neuroimaging of psychopathic traits. Front. Psych. 10:1027. doi: 10.3389/fpsyt.2019.01027, 32116828 PMC7016047

[ref43] Jurjako M. Malatesti L. Brazil I. A. (2019). Some ethical considerations about the use of biomarkers for the classification of adult antisocial individuals. Int. J. Forensic Ment. Health 18, 228–242. doi: 10.1080/14999013.2018.1485188

[ref44] Katzin S. Andiné P. Hofvander B. Billstedt E. Wallinius M. (2020). Exploring traumatic brain injuries and aggressive antisocial behaviors in young male violent offenders. Front. Psych. 11:507196. doi: 10.3389/fpsyt.2020.507196, 33192641 PMC7581682

[ref45] Kempes M. Berends I. Duits N. van den Brink W. (2019). Neurobiological information and consideration in Dutch pre-trial forensic reports of juvenile criminal offenders. Int. J. Forensic Ment. Health 18, 212–219. doi: 10.1080/14999013.2018.1525776

[ref46] Kiehl K. A. Anderson N. E. Aharoni E. Maurer J. M. Harenski K. A. Rao V. . (2018). Age of gray matters: neuroprediction of recidivism. Neuroimage Clin. 19, 813–823. doi: 10.1016/j.nicl.2018.05.036, 30013925 PMC6024200

[ref47] Krause L. Enticott P. G. Zangen A. Fitzgerald P. B. (2012). The role of medial prefrontal cortex in theory of mind: a deep rTMS study. Behav. Brain Res. 228, 87–90. doi: 10.1016/j.bbr.2011.11.037, 22155478

[ref48] Kuhn L. Choy O. Keller L. Habel U. Wagels L. (2024). Prefrontal tDCS modulates risk-taking in male violent offenders. Sci. Rep. 14:10087. doi: 10.1038/s41598-024-60795-z 38698192 PMC11066090

[ref49] Ligthart S. L. (2019). Coercive neuroimaging, criminal law, and privacy: a European perspective. J. Law Biosci. 6, 289–309. doi: 10.1093/jlb/lsz015, 31666970 PMC6813934

[ref50] Ligthart S. L. (2020). Freedom of thought in Europe: do advances in ‘brain-reading’technology call for revision? J. Law Biosci. 7:lsaa048. doi: 10.1093/jlb/lsaa048, 34221423 PMC8249165

[ref51] Ligthart S. L. (2023). Neuro-interventies in het sanctierecht: Over negatieve en positieve vrijheden. Boom Strafblad 1, 45–51. doi: 10.5553/BSb/266669012023004001007

[ref52] Ligthart S. L. Dore-Horgan E. Meynen G. (2023). The various faces of vulnerability: offering neurointerventions to criminal offenders. J. Law Biosci. 10:lsad009. doi: 10.1093/jlb/lsad009, 37168841 PMC10165894

[ref53] Ligthart S. L. Douglas T. Bublitz C. Kooijmans T. Meynen G. (2021). Forensic brain-Reading and mental privacy in European human rights law: foundations and challenges. Neuroethics 14, 191–203. doi: 10.1007/s12152-020-09438-4, 35186162 PMC7612400

[ref9002] Ligthart S. Van Oploo L. Meijers J. Meynen G. Kooijmans T. (2019). Prison and the brain: Neuropsychological research in the light of the European Convention on Human Rights. New Journal of European Criminal Law 10:287–300.

[ref54] Lisoni J. Miotto P. Barlati S. Calza S. Crescini A. Deste G. . (2020). Change in core symptoms of borderline personality disorder by tDCS: a pilot study. Psychiatry Res. 291:113261. doi: 10.1016/j.psychres.2020.113261, 32622171

[ref55] Marazziti D. Baroni S. Landi P. Ceresoli D. Dell’Osso L. (2013). The neurobiology of moral sense: facts or hypotheses? Ann. General Psychiatry 12:6. doi: 10.1186/1744-859X-12-6, 23497376 PMC3616987

[ref57] Meynen G. (2017). Brain-based mind reading in forensic psychiatry: exploring possibilities and perils. J. Law Biosci. 4, 311–329. doi: 10.1093/jlb/lsx006

[ref58] Meynen G. (2020). "Neurorecht: hoop of hersenschim?". Den Haag: Boom juridisch.

[ref59] Meynen G. (2022). “Legal insanity in the Netherlands: regulations and reflections,” in The Insanity Defence: International and Comparative Perspectives, eds. Mackay R. Brookbanks W. (Oxford: Oxford Monographs on Criminal Law and Justice), 274–294.

[ref60] Meynen G. Van de Pol N. Tesink V. Ligthart S. (2023). Neurotechnology to reduce recidivism: ethical and legal challenges. Handb. Clin. Neurol. 197, 265–276. doi: 10.1016/B978-0-12-821375-9.00006-2, 37633715

[ref61] Mia A. P. (2025). From brain evidence to neurorights: rethinking criminal responsibility in the age of neurotechnology. SSRN Electron. J.:5588370. doi: 10.2139/ssrn.5588370

[ref62] Molero-Chamizo A. Riquel R. M. Moriana J. A. Nitsche M. A. Rivera Urbina G. N. (2019). Bilateral prefrontal cortex anodal tDCS effects on self reported aggressiveness in imprisoned violent offenders. Neuroscience 397, 31–40. doi: 10.1016/j.neuroscience.2018.11.018, 30472431

[ref63] Moore M. (2022). Freedom of thought at the ethical frontier of law & science. Ethics Behav. 32, 510–531. doi: 10.1080/10508422.2021.1928500

[ref64] Morse S. J. (2005). Brain overclaim syndrome and criminal responsibility: a diagnostic note. Ohio State J. Crim. Law 3:379. Available online at: http://hdl.handle.net/1874/419290

[ref65] Morse S. J. (2018). “Neuroscience in forensic contexts: ethical concerns,” in Ethics Challenges in Forensic Psychiatry and Psychology Practice, ed. Griffith E. E. H. (New York Chichester, West Sussex: Columbia University Press), 132–157.

[ref66] Nadelhoffer T. Bibas S. Grafton S. Kiehl K. A. Mansfield A. Sinnott-Armstrong W. . (2012). Neuroprediction, violence, and the law: setting the stage. Neuroethics 5, 67–99. doi: 10.1007/s12152-010-9095-z, 25083168 PMC4114735

[ref68] Pickard H. Fazel S. (2013). Substance abuse as a risk factor for violence in mental illness: some implications for forensic psychiatric practice and clinical ethics. Curr. Opin. Psychiatry 26, 349–354. doi: 10.1097/YCO.0b013e328361e798, 23722099 PMC3907744

[ref69] Pugh J. (2018) Neurocorrective Offer. Treatment for Crime: Philosophical Essays on Neurointerventions in Criminal Justice

[ref70] Riva P. Gabbiadini A. Romero Lauro L. J. (2017). Neuromodulation can reduce aggressive behavior elicited by violent video games. Cogn. Affect. Behav. Neurosci. 17, 452–459. doi: 10.3758/s13415-016-0490-8, 28035636

[ref71] Riva P. Romero Lauro L. J. DeWall C. N. Chester D. S. Bushman B. J. (2015). Reducing aggressive responses to social exclusion using transcranial direct current stimulation. Soc. Cogn. Affect. Neurosci. 10, 352–356. doi: 10.1093/scan/nsu053, 24748546 PMC4350477

[ref72] Ryberg J. (2017). Neuroethics and brain privacy: setting the stage. Res. Publica. 23, 153–158. doi: 10.1007/s11158-016-9340-3

[ref73] Saladino V. Lin H. Zamparelli E. Verrastro V. (2021). Neuroscience, empathy, and violent crime in an incarcerated population: a narrative review. Front. Psychol. 12:694212. doi: 10.3389/fpsyg.2021.694212, 34393924 PMC8355490

[ref74] Schreier M. (2012). Qualitative Content Analysis in Practice. London: Sage.

[ref75] Schuwerk T. Schecklmann M. Langguth B. Doehnel K. Sodian B. Sommer M. (2014). Inhibiting the posterior medial prefrontal cortex by rTMS decreases the discrepancy between self and other in theory of mind reasoning. Behav. Brain Res. 274, 312–318. doi: 10.1016/j.bbr.2014.08.031, 25157431

[ref76] Sergiou C. S. Santarnecchi E. Romanella S. M. Wieser M. J. Franken I. H. A. Rassin E. G. C. . (2022). Transcranial direct current stimulation targeting the ventromedial prefrontal cortex reduces reactive aggression and modulates electrophysiological responses in a forensic population. Biological Psychiatry 7, 95–107. doi: 10.1016/j.bpsc.2021.05.007, 34087482

[ref77] Sergiou C. S. Tatti E. Romanella S. M. Santarnecchi E. Weidema A. D. Rassin E. G. C. . (2023). The effect of HD-tDCS on brain oscillations and frontal synchronicity during resting-state EEG in violent offenders with a substance dependence. Int. J. Clin. Health Psychol. 23:100374. doi: 10.1016/j.ijchp.2023.100374, 36875007 PMC9982047

[ref79] Specker J. Focquaert F. Sterckx S. Schermer M. H. (2018). Forensic practitioners’ expectations and moral views regarding neurobiological interventions in offenders with mental disorders. BioSocieties 13, 304–321. doi: 10.1057/s41292-017-0069-9

[ref80] Spreen M. Brand E. Ter Horst P. Bogaerts S. (2014). Handleiding en Methodologische Verantwoording HKT-R, Historisch, Klinische en Toekomstige – Revisie [Guidlines and Methodological Research of the HKT-R, Historical, Clinical and Future – Revision]. Groningen, The Netherlands: Dr. van Mesdag kliniek.

[ref9001] Steele V. R. Claus E. D. Aharoni E. Vincent G. M. Calhoun V. D. Kiehl K. A. (2015). Multimodal imaging measures predict rearrest Frontiers in human neuroscience 9, 425. 26283947 10.3389/fnhum.2015.00425 PMC4522570

[ref81] Susser D. Cabrera L. Y. (2023). Brain data in context: are new rights the way to mental and brain privacy? AJOB Neurosci. 15, 122–133. doi: 10.1080/21507740.2023.2188275, 37017379

[ref83] Tesink V. Douglas T. Forsberg L. Ligthart S. Meynen G. (2023). Neurointerventions in criminal justice: on the scope of the moral right to bodily integrity. Neuroethics 16:26. doi: 10.1007/s12152-023-09526-1

[ref84] Tortora L. Meynen G. Bijlsma J. Tronci E. Ferracuti S. (2020). Neuroprediction and AI in forensic psychiatry and criminal justice: a neurolaw perspective. Front. Psychol. 11:220. doi: 10.3389/fpsyg.2020.00220, 32256422 PMC7090235

[ref85] Trabucco F. R. (2023). Neurorights between ethical and legal implications. Cuad. Derecho Transnacional 15:750. doi: 10.20318/cdt.2023.7561

[ref86] Turnwald B. P. Goyer J. P. Boles D. Z. Silder A. Delp S. L. Crum A. J. (2019). Learning one’s genetic risk changes physiology independent of actual genetic risk. Nat. Hum. Behav. 3, 48–56. doi: 10.1038/s41562-018-0483-4, 30932047 PMC6874306

[ref87] Van Dongen J. D. M. (2020). The empathic brain of psychopaths: from social science to neuroscience in empathy. Front. Psychol. 11:493832. doi: 10.3389/fpsyg.2020.00695, 32477201 PMC7241099

[ref88] Van Dongen J. D. M. Haveman Y. Sergiou C. S. Choy O. (2025). Neuroprediction of violence and criminal behavior using neuro-imaging data: from innovation to considerations for future directions. Aggress. Violent Behav. 80:102008. doi: 10.1016/j.avb.2024.102008

[ref89] Verweij S. Tollenaar N. Teerlink M. Weijters G. (2021) "Recidive Onder Justitiabelen in Nederland." Utrecht: WODC.

[ref90] Zijlmans J. Marhe R. Bevaart F. Van Duin L. Luijks M. A. Franken I. . (2021). The predictive value of neurobiological measures for recidivism in delinquent male young adults. J. Psychiatry Neurosci. 46, E271–E280. doi: 10.1503/jpn.200103, 33844482 PMC8061739

